# Reducing sick leave, improving work ability, and quality of life in patients with mild to moderate Long COVID through psychosocial, physiotherapeutic, and nutritive supportive digital intervention (MiLoCoDaS): study protocol for a randomized controlled trial

**DOI:** 10.1186/s13063-023-07819-7

**Published:** 2023-12-08

**Authors:** Adrian Krotz, Nadia Sosnowsky-Waschek, Stephanie Bechtel, Christine Neumann, Monika Lohkamp, Gabor Kovacs, Bernd Genser, Joachim E. Fischer

**Affiliations:** 1 Center for Preventive Medicine and Digital Health (CPD), Division of General Medicine, Medical Faculty Mannheim, Heidelberg University, Heidelberg, Germany; 2 School of Applied Psychology, SRH University of Applied Sciences Heidelberg, Heidelberg, Germany; 3 School of Therapeutic Sciences, SRH University of Applied Sciences Heidelberg, Heidelberg, Germany; 4 Department of Applied Digital Product Development, SRH Berlin University of Applied Sciences, Berlin, Germany; 5 High5Data, Heidelberg, Germany

**Keywords:** Post-acute COVID-19 syndrome, Long COVID, Post-COVID condition, Work ability, Sick leave, Fatigue, Randomized controlled trial, Long-term follow-up, Digital intervention, Psychological support

## Abstract

**Background:**

Following ﻿SARS-CoV-2 infection, a relevant proportion of patients suffer from persistent or recurring sequela, even after initially mild primary illness. Many patients experience exhaustion and fatigue, rendering them incapable of working. Long COVID exerts a substantial burden on society and the healthcare system: at least 65 million people are currently affected worldwide. The underlying pathobiology is a complex derangement in several organ systems. To date, causal pharmaceutical therapies remain elusive. Waiting lists for specialist care are long. Rapidly scalable digital interventions offering support for the frequent subgroup of patients with mild to moderate impairment from Long COVID are urgently needed. The MiLoCoDaS study compares three intensities of a potentially rapidly scalable digital intervention aiming to accelerate recovery. The overall objective is to figure out if there is a difference in the effect sizes between these modalities.

**Methods:**

The online intervention uses a learning platform (LMS, TYPO3 framework) comprising 12 sessions of medical, psychological, physiotherapeutic, and nutritional content. The three modalities differ as follows: patient information only (sham intervention, control), information plus interactive digital workbook including practical exercises (digital intervention), and the digital workbook augmented by once-weekly online seminars and discussion groups (person and peer-contact). Eligible patients are 18–67 years old satisfying Long COVID diagnostic criteria. Patients are recruited through primary care physicians and randomly allocated. The primary endpoint is the number of sick leave days during the 6-month observation period; secondary endpoints are patient-reported symptoms, quality of life, and work ability. The study size provides a power of 80% at a type I error of < 0.05 to show an effect size of Cohen = 0.3 between the augmented and the sham intervention (*N* = 152 per arm, total accounting for attrition *N* = 600).

**Discussion:**

If one of the two interventions is superior to providing information alone, MiLoCoDaS would provide the starting point for a rapidly scalable digital intervention for the frequent and currently underserved patient group with mild to moderate impairment from Long COVID. Several caveats pertain to the heterogeneity of Long COVID manifestation and duration prior to inclusion. It is conceivable that the possible effect of the intervention may differ across subgroups. Therefore, a priori defined secondary analysis will be conducted.

**Trial registration:**

German Clinical Trials Register (DRKS) DRKS00028964. Registered on 24 August 2022.

**Supplementary Information:**

The online version contains supplementary material available at 10.1186/s13063-023-07819-7.

## Administrative information

Note: the numbers in curly brackets in this protocol refer to SPIRIT checklist item numbers. The order of the items has been modified to group similar items (see http://www.equator-network.org/reporting-guidelines/spirit-2013-statement-defining-standard-protocol-items-for-clinical-trials/).

**Table:** 

Title {1}	Reducing sick leave, improving work ability and quality of life in patients with mild to moderate Long COVID through psychosocial, physiotherapeutic and nutritive supportive digital intervention (MiLoCoDaS): study protocol for a randomized controlled trial.
Trial registration {2a and 2b}.	DRKS00028964 [German Clinical Trials Register (DRKS)], https://drks.de/search/en/trial/DRKS00028964 [registered on 2022-08-24]
Protocol version {3}	Version 2.3 of 2023-03-01
Funding {4}	This research is funded by the Ministry of Science, Research and the Arts of Baden-Württemberg (MWK, Baden-Württemberg, Germany). The funded framework project is entitled AMBIGOAL-ANCOR ("ANwendungserweiterungen COvid-19 Restitutionsphasen").
Author details {5a}	Krotz, A.: Medical Faculty Mannheim, Heidelberg University, Center for Preventive Medicine and Digital Health (CPD), Division of General Medicine. Principal Investigator. Sosnowsky-Waschek, N.: SRH University of Applied Sciences Heidelberg, School of Applied Psychology. Bechtel, S.: Medical Faculty Mannheim, Heidelberg University, Center for Preventive Medicine and Digital Health (CPD), Division of General Medicine. Neumann, C.: Medical Faculty Mannheim, Heidelberg University, Center for Preventive Medicine and Digital Health (CPD), Division of General Medicine. Lohkamp, M.: SRH University of Applied Sciences Heidelberg, School of Therapeutic Sciences. Kovács, G.: SRH Berlin University of Applied Sciences, Department of Applied Digital Product Development Genser, B.: High5Data, Heidelberg, Germany Fischer, J.E.: Medical Faculty Mannheim, Heidelberg University, Center for Preventive Medicine and Digital Health (CPD), Division of General Medicine. Primary Sponsor.
Name and contact information for the trial sponsor {5b}	primary sponsor: Fischer, J.E. (Joachim.Fischer@medma.uni-heidelberg.de)
Role of sponsor {5c}	The primary sponsor has lead responsibility and ultimate authority for the study design decision to submit the report for publication. He decided on the study group's proposals for conceptual adjustments, collection, management, analysis, and interpretation of data. The funder played no role in the study design, planning, collection, management, analysis, and interpretation of the data, nor in the writing of the report.

## Introduction

### Background and rationale {6a}

Following initial infection with SARS-CoV-2, a considerable proportion of patients suffer from a variety of persisting or de novo occurring symptoms, known as post-COVID-19 condition or Long COVID. Depending on the definition used, the unadjusted prevalence reported in the literature varies substantially [1–5]. The persistence of at least some COVID-19-associated symptoms is described for up to 60% of non-hospitalized patients or those with mild initial clinical course [6, 7]. While these early figures derived from infections with pre-Omicron variants may not reflect current prevalence, even under conservative assumptions, an adjusted prevalence of 12.7% among previous COVID-19 patients must be expected in the community setting [8].

The severity distribution appears to be skewed with a larger proportion of affected patients experiencing mild to moderate symptoms that impair work ability and quality of life, but do not render these patients fully incapable of daily life activities. However, the impact on work ability implies a substantial public health and economic burden for society, given the millions of people affected across Europe.

Long COVID comprises a staggering multitude of symptoms, e.g., fatigue, brain fog, anxiety, depression, shortness of breath, headache, cough, impaired sleep, dizziness, neck, joint, chest and general muscle pain, hair loss, and very often impaired daily activity [1, 4, 6–10]. Hence, Long COVID impairs cognitive and physical performance as well as quality of life. Particularly those with prolonged and severe fatigue, neurocognitive impairment, depression, or pain experience substantially impaired work ability [11]. An international study from 56 countries reported that about one in five individuals suffering from Long COVID symptoms were unable to work due to the illness, while almost half had to reduce their working hours [12]. The largest German statutory health insurance reported an average of 105 sick leave days per year (p.a.) for severe Long COVID patients compared to the population average of 14.6 sick leave days [13]. For Germany, the economic burden of lost production and the lost gross value added arising from incapacity to work is estimated as a combined national economic loss of approx. 350 € per day of sick leave [14]. Similar calculations for the entire US estimate the total economic burden of lost work due to Long COVID to 175 billion US$ annually [15].

Multiple possible pathways have been suggested to cause the unexplained symptoms of Long COVID, from the persistence of SARS-CoV-2 antigen or genetic material, immune dysregulation, reactivation of other latent viral infections to microvascular dysfunction, among others. These pathophysiologic studies have suggested biological pathways as potential targets in therapeutic trials. Both repurposed existing medicines and novel therapeutics require formal scrutiny prior to widespread use in the affected population. Hence, at the design of our study, no clear consensus has emerged regarding the best pharmacotherapeutic options in patients with mild to moderate Long COVID [16–18].

Since there is currently still no pharmaceutical cure for Long COVID available, the focus of care hinges merely on supportive and symptomatic therapies. However, the large number of Long COVID patients contrasts the scarcity of specialized outpatient clinics. Naturally, preference in waiting lists is given to the most severe cases over patients with mild to moderate symptoms. Facing this challenging situation, we reasoned that—like in adjuvant cancer treatment—psychosocial support, nutritional advice, and physiotherapeutic exercises that enhance one’s own well-being and body perception might support accelerated recovery. To maximize reach and scalability, we chose to design a digital online intervention.

Digital online interventions had long been established before the COVID-19 pandemic. Substantial progress has been made from early attempts using computer-tailored smoking cessation messages in family practice in the 90s of the last century to systematic work aimed at improving effect sizes of digital therapeutics during the second decade of this century [19–22]. Emerging discussions now relate to the design of the appropriate control condition—as considered in this study design, to the effect-size heterogeneity depending on population, target disease, and modalities of the intervention [21, 23]. Scientific knowledge gaps still regard the appropriate application of the intervention, as shown in a recent systematic review including 19 reports from randomized controlled trials on online psychosocial interventions for improving mental health in patients during the COVID-19 pandemic [23]. It was found that in principle, online psychosocial interventions ameliorated anxiety (standardized mean difference (SMD) = − 0.78), depression (SMD = − 0.80), or insomnia (SMD = − 0.19) in the public. However, subgroup analyses that trial results depended on the modality, duration, setting, and type of intervention, hampering any conclusion regarding the specific population of patients with mild to moderate Long COVID [23]. This provided the rationale for our study design of delivering 12 different sessions with meticulous tracking of meta-data as to utilization and duration of engagement even on the micro level of individual elements offered within each of the 12 modules developed.

Initially, most digital interventions during the pandemic simply transitioned from direct person encounters to telemedical provision of care, leaving a scarcity of work building upon the already existing large body of theoretical insights into the design of digital mental health interventions [24]. For example, current state-of-the-art discussions address the augmentation of digital mental health interventions (DMHIs) by human support. A recent systematic review concluded that human-supported DMHIs may be more effective than unsupported DMHIs for individuals with elevated mental health symptoms, lending support to our hypothesis that human support by online group seminars might enhance effect sizes [22]. Finally, a survey including 740 occupational therapists from 69 countries related to COVID-19-specific occupational therapy found that respondents provided various interventions to support recovery in desired and needed daily activities [25]. Most interventions focused on fatigue management, cognition, relaxation, self-management, environmental adaptation, and mental health. The authors emphasize that fatigue, breathlessness, memory and concentration problems, and pain often disrupt daily occupational functioning. This is in line with our design to address different psychosocial health problems during the 12 sessions and develop goal-oriented, person-centered approaches to address coping with the impairments in occupational and daily functioning.

While digital interventions have shown small to moderate effect sizes in the treatment of depression [26], there is still a controversy about the most efficient mode of delivery. A recent review suggests that personally supervised or guided interventions may yield larger effect sizes than pure interactive digital self-learning modalities in comparison to information alone [27]. The advantage of pure digital intervention is the much simpler scalability. In addition to the requirement of therapeutically addressing a complex, multimodal disease pattern for a large amount of patients, ascertaining access to adequate care in rural areas represents an additional challenge. Finally, beyond accessibility and rapid availability, the intensity and pace of an intervention should be individually tailored to the patient’s current performance level. A digital treatment concept satisfies those criteria and is location-independent and scalable, thus offering a potential option for those patients on the moderate to mild spectrum of Long COVID disease severity. Thus, we designed an online learning management system (LMS) based on TYPO3 as the digital delivery platform.

The present “mild to moderate Long COVID digital intervention study” (MiLoCoDaS) aims to elucidate which alternative modalities of a tailored educational digital intervention show the largest efficacy. The study compares three modalities, where information alone serves as control, and digital interactive intervention or digital interactive plus video group sessions as the two modalities of differing intensity. The content was assembled equally suitable for patients with already manifested Long COVID disease (treatment criterion) as for patients currently suffering from COVID-19 (prevention criterion). Given the large number of patients suffering from mild to moderate Long COVID, efficacy alone should not be the single criterion for further planning, but also the potential scalability. While we are well aware that the quality of life and ability to manage daily activities are potentially the most relevant endpoints from the patient's perspective, we deliberately chose the number of sick leave days as primary endpoint. The rationale for this is that each lost day can at least for Germany be easily quantified from well-defined economic numbers, rendering future justification of the more expensive intense intervention much easier. This rationale is equally applicable to all countries where there is a legally regulated payment of wages in case of illness and structured statistical data on the socio-economic costs of sick days as well.

### Objectives {7}

The primary aim of this study is to determine whether two different intensities of a 12-week online-based tailored educational digital intervention program for Long COVID patients (and currently infected prevention patients) provide a larger benefit than well-presented digital information alone on the primary endpoint days of sick leave accumulated over a 6-month period. If patients are in a structured employment reintegration program, reintegration days and employment percentage (hours compared to full time) will be used.

The secondary objectives are change in symptom severity or frequency, quality of life, work ability and work productivity, participation and dropout rates, and the usage behavior of the LMS content during the 6-month observation period.

### Trial design {8}

MiLoCoDaS is designed as a prospective, three-arm, randomized, stratified, controlled superiority trial, with an equal allocation ratio of 1:1:1.

A checklist according to the recommendations for interventional trials (SPIRIT) is attached as supplementary material (see Additional file 1) [28].

## Methods: participants, interventions, and outcomes

### Study setting {9}

The MiLoCoDaS study is conducted in Germany and was designed based on specifications of the local healthcare system and labor law, such as:

- In Germany, some general practitioners (GP) form networks in the sense of a cooperative. This enables their practices to centralize various administrative tasks, public relations, postgraduate training, and research involvement, to facilitate cross-sector collaboration and to apply uniform quality standards.
- If an employee in Germany is unable to work due to illness, she is obliged to obtain a medical certificate of incapacity for work from her GP and submit this to the employer. The GP documents the sick leave days and reports them to the statutory health insurance. The mandate for this arises from the German sick leave legislation. Provided sickness is medically certified, employees are entitled to 6 weeks of continuous sick leave salary paid by the employer, thereafter for a substantial proportion of sick leave remuneration paid by the statutory health insurance for up to 180 total sickness days.
- In Germany, GPs can prescribe some apps or similar products (so-called digital health applications (“DiGA”)). Their use is then paid for by the statutory insurer.

**Fig. 1:**
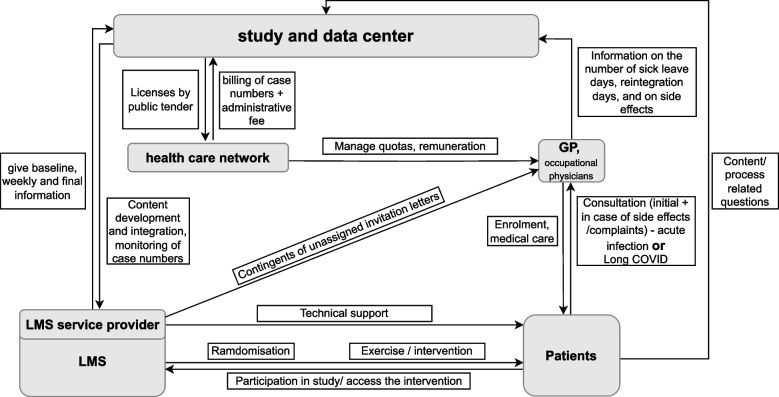
Network structure of stakeholders; LMS, learning management system; GP, general practitioner

The coordinating study center for MiLoCoDaS is the university medical center at the Mannheim Medical Faculty, Heidelberg University. From there, cooperation takes place via different primary care networks and their affiliated GP’s practices in the entire German state of Baden-Württemberg and other selected areas in Germany. Patients are included in the study directly by their GP, who after obtaining informed consent provides participants with the personalized access code to the online intervention platform. Each participating GP is responsible for the medical care during the study period of the recruited patients. The GP is likewise responsible for documentation and data reporting (incl. primary objective data) to the study center. The study interventions are provided exclusively online. Alternatively, an occupational physician can undertake the GP’s function. For example, during the legally mandated reintegration program for long-term sick leave in Germany, occupational health physicians often coordinate efforts. For occupational physicians, however, exactly the same processes and rules apply. The network structure of stakeholders is shown in Fig. 1.

As the abovementioned specifications of the local healthcare system in Germany only affect organizational purposes in the trial setting, the results of the trial can nonetheless be adapted to different organizational structures worldwide.

### Eligibility criteria {10}

We originally intended to differentiate between recent COVID infection (digital intervention as prevention) and manifest Long COVID (therapeutic application). However, for process simplicity during recruitment, we selected similar criteria:

Inclusion criteria (treatment): (a) SARS-CoV-2 infection at least 3 months ago and (b) ICD-10 code U08.9 or U09.9 given or persistent/recurring symptoms according to the WHO clinical case definition of post COVID-19 condition by a Delphi consensus.

OR:

Inclusion criteria (prevention): (a) PCR or antigen test to detect SARS-CoV-2 infection at least 7 days prior to the consultation with (b) persisting incapacity for work.

Additional common inclusion criteria (both paths): (c) Patient is between 18 and 67 years old (legal age of majority and retirement age in Germany) and (d) anamnestic score for general health and daily activities on a numerical analog scale (0–10) is at least 2 points worse than before the SARS-CoV-2 infection.

Exclusion criteria:

- Hospitalization due to COVID-19
- Repeated treatment by a specialist (excluding consultations for diagnostic purposes) due to COVID-19-related complaints
- Strong spoken and written language barrier
- Patient does not have access to a terminal device with an Internet connection
- Patient is currently suffering from symptomatic cancer
- Patient is currently undergoing chemotherapy
- Existing neurological disease affecting cognition (esp. stroke, dementia, etc.)
- Current symptomatic psychiatric illness, if first diagnosed in the last calendar year and not caused by COVID-19

## Who will take informed consent? {26a}

After a patient has been assessed as eligible based on the abovementioned criteria, the GP provides detailed information about the study objectives, procedures, and potential risks and benefits. Participants are also handed a detailed written patient information document and have the opportunity to ask their GP any questions related to the study. Thereafter, the GP obtains written consent and keeps the record safe in accordance with data protection law requirements.

### Additional consent provisions for collection and use of participant data and biological specimens {26b}

In addition to the general consent to study participation, participants release their GP by written consent from medical confidentiality for the purpose of transferring their data to the study center.

When participants first register on the intervention LMS, they receive detailed information about data privacy according to the General Data Protection Regulation (GDPR). Their consent to data processing in the LMS is obtained digitally during the registration process and stored in the LMS database in accordance with the legal requirements.

### Interventions

#### Explanation for the choice of comparators {6b}

The control group follows the same overarching study procedures (i.e., informed consent) and has access to the LMS just like the intervention groups. Potential effects of the study procedures or the LMS environment therefore also affect the control group. However, the control group only receives professionally prepared information but no interactive didactic structure nor any interactive/functional elements within the LMS. There is no coaching and no contact person for content-related questions. The LMS content accessible for the control group is similar to an extended fashion of digital information becoming increasingly available from statutory health insurance websites. Laypersons could assemble the information by intensive literature or Internet search. Hence, the control group thus receives a sham intervention with no expected effect beyond what can currently be found by diligent Internet search.

Nevertheless, the control group is provided with sufficient information and video material to ascertain that the minimum processing and material exposure times explained in the patient information and documents consent remain plausible to avoid suspicion of a placebo intervention in the patients. The control group is also subject to the same adherence mechanisms as the intervention groups. At the study termination, we will inquire patients about their best guess whether they were included in the control or intervention group.

#### Intervention description {11a}

All interventions (including control) are applied through the MiLoCoDaS LMS, a digital online learning platform based on TYPO3. After registration, answering a baseline questionnaire, and automated randomization, participants get access to the content of one of the intervention groups or the control group.

##### High-intensity intervention

Participants are provided a 12-week closed-group online workshop (webinar) combined with a 12-week interactive digital self-study course that delivers similar and complementary content. The webinar consists of 12 sessions, each approximately 45–90 min, once a week on fixed dates, with 15–25 people per group. The groups, webinar coaches, and dates are constant. Static information materials are additionally provided. This static information material is defined as having not been didactically optimized and laypersons could find the information themselves with intensive literature or Internet search.

The webinars and the self-study course are structured into 12 modules, which build upon each other in terms of content and are completed in chronological order. Each module addresses a specific topic based on typical complaints of patients with post-COVID-19 disease. Central topics among others include coping with everyday life, structuring the day, increasing or pacing physical activity and the ability to relax, coping with pain, promoting acceptance and a positive basic attitude, improving cognitive performance, and the ability to regulate emotions.

The webinars are facilitated by psychological coaches. Only one specific module (Module 2), which deals exclusively with medical information and context (such as symptom correlations or pathophysiological explanations), is facilitated by licensed physicians who have been researching Long COVID since the disease emerged. The psychological coaches were selected and trained by the psychological head investigator. Decisive qualifications were previous academic achievements, especially in the field of clinical and health psychology, additional qualifications (e.g., in the form of additional certificates such as “Coaching and Counselling,” “Conversation Management,” etc.), and previous experience in leading therapeutic groups (e.g., through relevant internships). These coaches then received five standardized and interactive training sessions of 2–3 h each in group format from the psychological head investigator. The coaches were introduced to the content, objectives, and didactic concept of the LMS and given precise instructions on how to structure the online sessions and how to deal with potentially difficult discussion situations. Each coach received a detailed course manual with a precise description of the exercises to be performed, their sequence, duration, details of the discussions to be held, reflections, and psychoeducational input. In addition, they received further training and a compendium on the status quo of knowledge about Long COVID. All irregularities in webinars, whether communicative, organizational, professional, emotional, etc., are discussed with the psychological head investigator in regular supervision sessions.

The digital self-study course has been refined by media-didactic experts and uses elements and gamifications such as quizzes, memory trainings, interactive pain maps, graphical visualization, social network maps, responsive self-reflection tools, and others. Furthermore, all figures in illustrations were depicted in a gender-neutral form and with varying ethnic characteristics to avoid bias in the perception of content by association with a social role model or stereotypes. The media-didactic optimization was conducted in order to make the LMS as user-friendly and easy to understand as possible, as well as to make the overall user experience engaging. It was also intended to ensure accessibility. All of these measures are supposed to increase the learning success of the participants and serve to strengthen adherence. Therefore, an experienced professor of applied digital product development, himself a media pedagogue and communication designer, and his team served as media-didactic experts. This provided many years of experience in interaction design, digital product development, and the development and optimization of content management systems, mass customizing platforms, and e-learning systems.

The provision of course exercises is partly adapted according to the answers in the baseline questionnaire. Depending on indications of special disease manifestations (e.g., chronic fatigue syndrome), some exercises are only offered in a limited version or with additional instructions. At the beginning, only the first-course module can be accessed. On the day of the respective webinars, the corresponding modules are unlocked for the participants.

The content of both the webinars and the self-study course is interdisciplinary and provides evidence-based information, recommendations, and exercises from medicine, psychology, physiotherapy, and nutritional sciences. If participants suffer from a particular medical condition that renders specific exercises to be recommended only in a limited or reduced version (i.e., physiotherapeutic exercises), the LMS content automatically adapts according to the given answers in the medical symptom screening. Participants are able to contact their coach via the integrated chat function, if they have any questions or require further advice. In addition, technical support is available by email.

##### Moderate intensity intervention

The same 12-week interactive self-study course is available as in the high-intensity arm with all features, except for the webinars. Again, only the first module is activated in the self-study course at the beginning. In this arm, however, participants can unlock the following modules independently by completing the previous one. They therefore determine the learning rhythm completely on their own. The participants are advised several times during the course that the modules have been designed in such a way that the optimum processing time for each module is about 1 week. Static information material is equally provided as in the high-intensity arm. Since participants are not assigned to a personal coach without the webinars, they direct their inquiries to a coach-supervisor via the chat function to ask for advice. Requests on psychological topics are answered by the psychological head investigator, while medical questions are answered by a physician of the study team.

The coach-supervisor also leads the quality assurance and supervision sessions in the high-intensity intervention. For specific questions, the coach-supervisor seeks the advice of a qualified specialist from the study team (physician, physiotherapist, etc.).

##### Control

Only content that participants could have found without professional guidance should be available here. For ethical reasons, a quality check was carried out to ensure that all information provided is basically correct and does not conflict with the principles of evidence-based treatment. Participants therefore receive 12-week access to a reduced version of the self-study course containing only the static information materials. All interactive, responsive, or didactically optimized content is not offered. At the beginning, again only module 1 is unlocked and the activation of further modules is equivalent to the moderate intensity arm. The chat function, coach support, or other didactic interaction are not available to participants in this arm.

#### Criteria for discontinuing or modifying allocated interventions {11b}

No changes to the assigned interventions are intended. However, all exercises shown in the program are voluntary and can be done or skipped by the participants individually. Thus, less intervention components deemed by the participant to be less beneficial can be excluded individually.

The attending GP may exclude participants from further participation at any time for medical reasons. The assessment of reasons (e.g., worsening disease) is the exclusive responsibility of the GP.

Participants themselves can terminate their participation at any time by revoking their consent.

Any of the following situations during the study period will also lead to discontinuation:

- Hospitalization due to COVID-19 or post-COVID-19-disease.
- Referral to long-term specialist outpatient treatment due to COVID-19 or post-COVID-19 disease.

#### Strategies to improve adherence to interventions {11c}

For monitoring adherence and participation indices, the LMS provides a dashboard that can only be accessed by the study management team. It allows real-time monitoring of participation numbers and group distributions.

Several measures have been taken to increase adherence.

##### Starter kits

All participants receive a starter package as a gift upon joining the study. The package consists of branded carrier bags containing a ball pen, a keychain, and a notebook that participants can use to work on exercises during the program.

##### Reminder emails

Participants receive a reminder email at defined events within the patient journey, in case of inactivity or if they miss a checkpoint in the process. Specifically:

- In case of an incomplete registration process.
- Appointment reminders on webinar days.
- In case of inactivity (7 days without interaction in the LMS).
- When there is a new system message or chat message in the LMS inbox.
- In case of non-response to a mandatory questionnaire.

##### Incentives

Among all participants who have completed 80% or more of the evaluation measures, €25 e-gift vouchers will be raffled (about 1 in 3 chances).

##### Feedback on health determinants

There are various health trackers within the LMS platform that participants can use to monitor their progress throughout the program. In addition, after each completion of the short weekly symptom questionnaire, participants receive direct feedback as a graphical visualization of their scores on multiple determinants of health in comparison to the previous week. This feature is also available to the control group and exceeds what participants would find on other websites or on the Internet.

#### Relevant concomitant care permitted or prohibited during the trial {11d}

All concomitant treatments or interventions that match the exclusion criteria described in {10} are prohibited during the trial. The same applies to interventions directly caused by any of the exclusion criteria, e.g., psychiatric therapy due to a current psychiatric illness. Other treatments are accordingly allowed.

#### Provisions for post-trial care {30}

Follow-up care after the trial is guaranteed as follows:

By involving the treating GP as the study physician, follow-up treatment is ensured via the usual care with full transparency about the interventions carried out.

A trial participant insurance policy was contracted for all participants.

### Outcomes {12}

#### Primary outcome

Primary outcome is the cumulative sum of sick leave days in patients during the period from enrollment to 6-month follow-up. We compare the high-intensity intervention versus moderate-intensity intervention versus control group. In Germany, every employee requires a medical sick leave certificate. The medical sick leave certificate entitles to 6 weeks of continuous payment of the salary by the employer. Thereafter, for additional 180 days, the statutory health insurance provides 80% of the net salary. Law mandates that employers offer employees with sick leave of longer than 6-week duration the opportunity to participate in a structured employment reintegration program (BEM). In such programs, patients are attempted to be reintegrated into their former jobs in agreement with the occupational health physician, the employer, and the employees’ representative board. The usual process is to gradually increase the weekly job hours and job requirements expressed as a percentage of full employment. Therefore, if a patient takes part in such a program, the sum of days that the participant has spent within an integration percentage stage is used as the primary outcome instead (e.g., 10 working days at 50% equals 5 days on full sick leave). In both cases—sick leave days and reintegration days—the data is reported directly to the study management by treating GPs via their health care networks at once at the end of the trial.

#### Secondary outcomes

Secondary outcomes are change in quality of life, work ability, work productivity, and symptom occurrence and severity. These will be collected via questionnaires using patient-reported outcome measures (PROMs, see below). Participants complete comprehensive surveys at enrolment (T0 questionnaire, approx. 20 minutes completion time, baseline values), after finishing the 12 modules of the intervention period (T2 questionnaire, approx. 10-min completion time, after 3 months) and as mid-term follow-up 6 months after enrolment (T3 questionnaire, approx. 10-min completion time). In addition, a short questionnaire is collected weekly (T1 questionnaire, details see below). T0, T2, and T3 assess the following dimensions of health: health-related quality of life (mental and physical), physical complaints, depression, anxiety, burnout, pain, social support, physical fitness, shortness of breath, sleep quality, and work ability. Current medication, vaccination status, concentration issues, and individual COVID-related medical history as possible influencing parameters are collected at T0. The T0 questionnaire also obtains indicator scores for disease courses or symptoms which require adjusted specific module components. The disease courses or symptoms considered are post-exertional malaise (PEM), myalgic encephalomyelitis/chronic fatigue syndrome (ME/CFS), and orthostatic intolerance (OI). If required on the basis of the specific score of a participant, the LMS content is automatically adapted for high-intensity or moderate-intensity physical exercises within the intervention.

Once weekly during the intervention period, participants complete a short questionnaire (T1.1–T1.12) of 5-min completion time. This questionnaire offers short questions on current symptoms, well-being, and again work ability. As a direct feedback, participants receive a visualization in the form of a radar chart comparing their current values to the data of the previous week.

Furthermore, participants of the high-intensity and moderate-intensity groups have the possibility to report on how they are feeling in terms of energy level, overall health, pain, mood, number of enjoyable activities, and exercise level in visual analog scales via the LMS as often as they like during the intervention period. This tracker data is presented to the participants as a progress graph line chart.

The high rate of sick leave days in Long COVID patients could be directly related to the variety and severity of symptoms that patients experience [12, 29]. An improved ability to work could therefore be seen as an indicator for an improvement in the activities of daily life and the symptoms.

Further secondary outcomes are participation and dropout rates, including the exact dropout points during the process (directly derived from the user data of the LMS). Finally, we obtain meta-data on the use of resources, particularly the overall usage statistics of the LMS (e.g., which pages and multimedia content were accessed and how often, which bookmarks were set, which exercises were completed repeatedly and how often, how many reminder mails had to be sent and when).

At the end of the trial, all the secondary outcomes data described is exported at once by the provider of the LMS and sent to the study data management after appropriate pseudonymization.

Possible side effects are also recorded as secondary outcomes. These are reported directly and anonymously by the respective treating GP to the study management team. These reports include the methods used for diagnosis of side effects.

### Participant timeline {13}

**Fig. 2:**
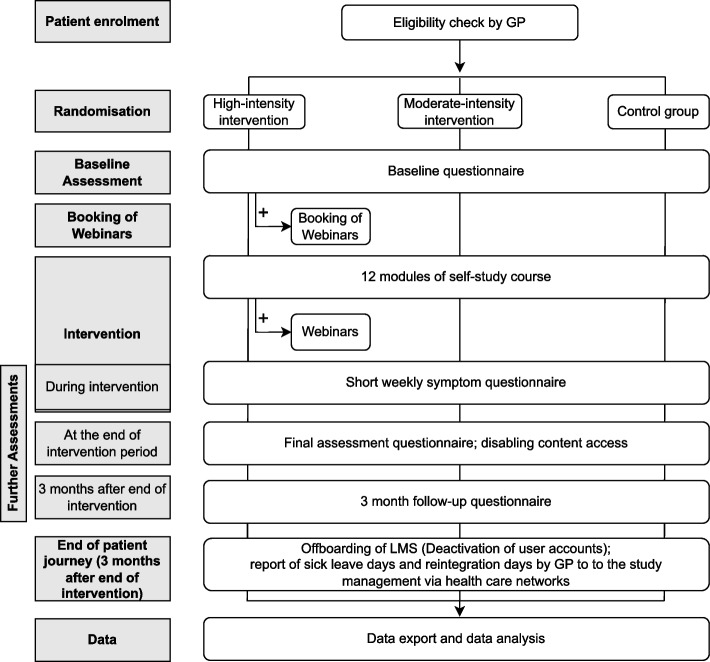
Patient journey and intervention assessment timeline; LMS, learning management system; GP, general practitioner

**Fig. 3:**
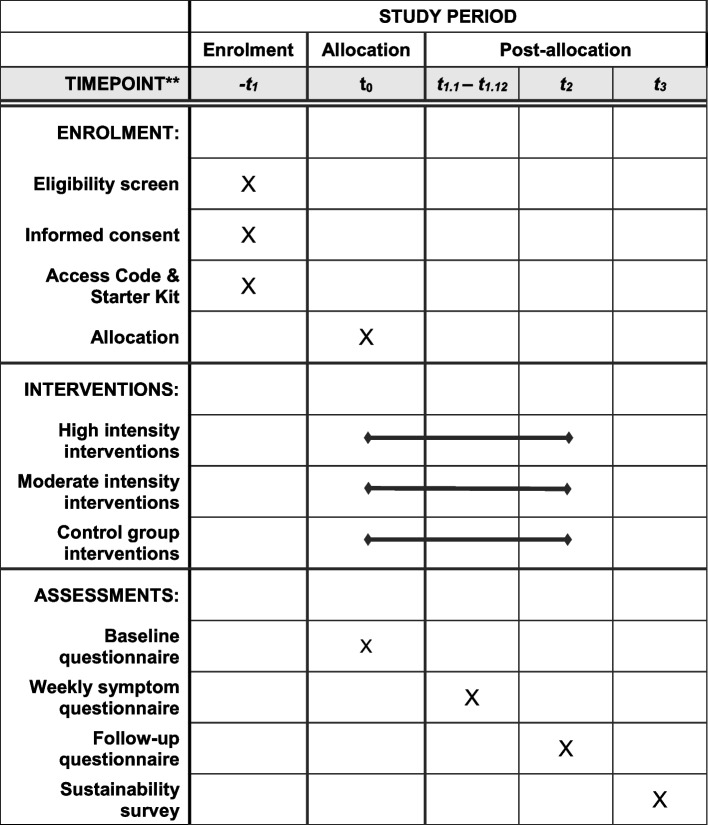
Schedule of enrolment, interventions, and assessments following the SPIRIT guidelines

The participant timeline is shown in Fig. 2. An alternative depiction following the SPIRIT schedule recommendation is shown in Fig. 3.

### Sample size {14}

The sample size was calculated for a randomized single-factor ANOVA design with 3 groups without clustering, assuming 30 days of illness (standard deviation of 10 days) during 180 days of follow-up in the control group and a reduction by 4 days for the high-intensity intervention (effect size of Cohen’s *d* = 0.4) and a reduction by 1 day in the medium intensity group. Such reduction was deemed by occupational health physicians as well as general practitioners as clinically highly relevant. Compared to the control group, a power of 80%, a type 1 error probability of less than 5%, and a dropout rate of 25% result in a required sample size or *N* = 151 per group. Thus, we aim to include at least *N* = 453 patients. In order to be able to exclude possible correlations due to socio-demographic variables corresponding to the location of participating practices (in particular rural doctors versus urban infrastructure), the target size is an increased sample size of 600 patients in total (*N* = 200 patients per study arm).

### Recruitment {15}

Patients will be recruited by health care networks and their affiliated general practitioners (GP) through various means, including (1) face-to-face invitations; (2) phone invitations; (3) promotional materials such as posters, leaflets, and brochures; (4) press releases and newspaper articles; (5) promotion through project website; and (6) promotion through social media. In addition, patients can be recruited by occupational physicians of different companies which will allow us to recruit a heterogeneous group covering a broad spectrum of professions.

### Assignment of interventions: allocation

#### Sequence generation {16a}

Blocked stratified randomization will be used to assign participants to the randomization groups. Strata have been defined by age, gender, type of employment (brain worker/physical worker), and status of disease (acute or Long COVID). Randomization sequence has been generated by the statistical software package STATA, function STRATA rand (StataCorp. 2019. Stata Statistical Software: Release 16. College Station, TX: StataCorp LLC).

#### Concealment mechanism {16b}

The allocation table is stored in the LMS and is queried by an automated script during the registration process. The stratification variables are known, because they are collected via the T0 questionnaire which takes place before the randomization process. In concrete terms, this means that after participants have registered in the LMS with their code, they complete the T0 questionnaire and are then automatically assigned to an intervention group. Thus, additional measures to establish the allocation sequence are not necessary, as no active action is required due to the automatism. Likewise, no measures to conceal the sequence are necessary due to the automation.

#### Implementation {16c}

The computer-generated allocation sequence was transformed into a randomization reference table for each stratum stored in spreadsheet calculation software. Patients register themselves in the LMS. Patients are then sequentially selected for randomization and classified to strata based on the stratification variables. The allocation number is read sequentially from the reference table for the respective stratum.

### Assignment of interventions: blinding

#### Who will be blinded {17a}

Participants themselves are blinded. They only receive the information that they have been assigned to an intervention group called “A,” “B,” or “C.” The participants do not know which measures are possible. They also do not know which intervention portfolio is associated with which letter. GPs are blinded in the way that they are not told which group a patient has been allocated to.

The study management and data analysts themselves are generally not blinded. However, they do not have direct access to participant data and participants during the trial period, and the evaluation is done on aggregated data once the study is completed.

#### Procedure for unblinding if needed {17b}

A process for unblinding is not envisaged, as no incidental diagnoses or other realistic events requiring immediate action by the participant are conceivable in the given conception. Nor would legal demands be likely to make this necessary.

### Data collection and management

#### Plans for assessment and collection of outcomes {18a}

##### Primary outcome

GPs document sick leave days and BEM days in patient records as part of their normal work outside the study. At the end of the trial, the GP totals these days per study participant she enrolled for a period of 6 months counting from the day of registration in the LMS. This registration is defined as the start of the intervention. The GP then writes the accumulated days into a pseudonymized list of her patients and transmits this file to the study center via an end-to-end encrypted virtual data safe access. This simple procedure reduces the workload for GPs and eliminates the need for special instruction.

##### Secondary outcomes

All questionnaires conducted in the trial are collected via integrated html-based forms within the LMS. All data collected via the LMS is stored in the related sql database.

Various dimensions of health are assessed via the PROM-based T0 to T3 questionnaires:

*Health-related quality of life (mental and physical)* is measured via short-form 12 (SF-12). The questionnaire consists of 12 items on general health perception (1 item), physical health (2 items), limited physical role function (2 items), physical pain (1 item), vitality (1 item), mental health (2 items), limited emotional role function (2 items), and social functioning (1 item).

The royalty-free version 1 of the questionnaire was used. By weighting and dependent calculation of the individual values, two scores on physical and mental health-related quality of life are generated according to a complex formula. In this trial, the values are not weighted with the orthogonal factor loadings suggested by the test authors, but with oblique factor loadings. It has been shown that this leads to more consistent results, since the calculation includes an interdependence of physical and mental quality of life [30]. Except for these adjustments, the SPSS rule is used for scoring as suggested in the test manual. The SF-12 is part of the T0, T2, and T3 questionnaires.

*Screening for depression* is carried out by a 9-item PHQ-9 subgroup of the patient health questionnaire family. This subgroup constitutes the depression module and can be used as an independent score to screen for depression. It measures common depression symptoms over a 2-week reference period via 4-point Likert scale with response categories ranging from “Not at all” to “Nearly every day”. Between 0 and 3 points are given for each answer and added up unweighted. The resulting sum score thus ranges from 0 to 27, with higher scores indicating more severe depression symptoms. The PHQ-9 shows good psychometric properties as a screening for depression when using the summed-item method [31, 32]. The PHQ-9 is part of the T0, T2, and T3 questionnaires.

*Physical complaints* are surveyed via the patient health questionnaire 15 (PHQ-15). The questionnaire consists of 15 items on the severity of the most common somatic symptoms. Each item has the 3 response categories “Not bothered at all” to “Bothered a little” to “Bothered a lot” asking about the symptom burden within the last 4 weeks. One of the mentioned symptoms refers to menstrual problems, so this is automatically not presented to male participants. Non-binary participants are presented with the item. In this trial, the PHQ-15 is not evaluated as a score, but as a distribution of symptom frequencies and symptom severity for each symptom recorded. Two of the items (concerning sleep problems and lack of energy) are also part of the PHQ-9. To calculate its score correctly, these two items are asked for a 2-week reference period like in the PHQ-9 instead of a 4-week reference period. The PHQ-15 is part of the T0, T2, and T3 questionnaires.

*Anxiety* is measured via the generalized anxiety disorder questionnaire (GAD-7). The questionnaire consists of 7 items on anxiety symptoms over a 2-week reference period via 4-point Likert scale with response categories ranging from “Not at all” to “Nearly every day”. Between 0 and 3 points are given for each answer and added up unweighted. The resulting sum score thus ranges from 0 to 21, with higher scores indicating higher anxiety. The GAD-7 as a self-report instrument for generalized anxiety disorder has good reliability and construct validity with an optimized sensitivity of 89% and specificity of 82% [33]. There is a high correlation between severity in the GAD-7 and disability scores [34]. The GAD-7 is part of the T0, T2, and T3 questionnaires.

*Burnout (personal, work-related)* is measured via Copenhagen Burnout Inventory (CBI). It divides the symptomatology into personal, work-related, and client-related burnout. Each scale can be calculated independently. All 3 scales show good reliability levels [35]. The questionnaire is administered to check the successful differential diagnosis between burnout and Long COVID. In addition, a possible deviating effect of the MiLoCoDaS intervention plan on varying burnout scores might be discovered. Since the client-related burnout does not essentially affect the research question, this subscale was not included in the survey for reasons of test economy. The chosen subscales consist of 13 items on burnout symptoms using 5-point Likert scales with response categories ranging from “Never / very rarely” to “Always” or from “To a very low degree” to “To a very high degree” respectively. Between 0 and 4 points are given for each answer and added up unweighted. In the resulting sum score, higher values are indicating higher burnout probability. The CBI is part of the T0, T2, and T3 questionnaires.

*Pain* is measured via an adaptation of the Cornell Musculoskeletal Discomfort Questionnaire (CMDQ). In this trial, the CMDQ is not evaluated as a score, but as a distribution of pain locations, frequency, and severity among Long COVID patients. In the first step, the particular pain localization is asked using a suggestion list. Then, the participants rate the pain intensity per previously named region on a numerical analog scale of 1–10, with higher values representing more pain. At last, participants rate the impairment of their usual activities by the reported pain. The German version of the CMDQ shows good psychometric properties [36]. The CMDQ is part of the T0, T2, and T3 questionnaires.

*Social support* is measured via the ENRICHD-Social-Support-Instrument (ESSI), the social support subscale from the Enhancing Recovery in Coronary Heart Disease study (ENRICHD) questionnaire. The questionnaire consists of 5 items on individual availability of social support using a 5-point Likert scale with response categories ranging from “None of the time” to “All of the time”. Between 1 and 5 points are given for each answer. Items are then added up for an unweighted sum score, with higher values indicating greater social support. The ESSI shows good psychometric properties including the German adaptation [37, 38]. The ESSI is part of the T0, T2, and T3 questionnaires.

*Physical fitness* is measured via the 12-item short form of the questionnaire for the assessment of physical function status (FFB-MOT). The questionnaire consists of 12 items on physical activities related to everyday life using a 5-point Likert scale with response categories ranging from “I cannot do this activity” to “I do not have any problems.” Between 1 and 5 points are given for each answer. Items are then added up for an unweighted sum score, with higher values indicating better physical fitness. The FFB-MOT shows good psychometric properties for general populations [39]. The FFB-MOT is part of the T0, T2, and T3 questionnaires.

*Sleep quality* is measured via 5 mixed items on overall assessment of sleep quality, daytime sleepiness, problems falling asleep and staying asleep, and restfulness of sleep. Response categories use 5-point and 6-point Likert scales on quality (overall assessment) and frequency (other terms). Items on sleep problems and restfulness are inverted, so that higher values indicate better sleep. Afterwards, the items’ values are harmonized to a range of 0–100 to equalize the different response scales. Then, an unweighted mean is calculated. A root-based transformation is performed to compensate for ceiling effects and finally standardized to a mean of 66 and standard deviation (SD) of 15. Questions on sleep quality are part of the T0, T2, and T3 questionnaires.

*Work ability* is measured by an abbreviated assessment based on the Work Ability Index (WAI). The original version consists of 7 indicators on (WAI 1) Current work ability compared with the lifetime best, (WAI 2) Work ability in relation to the demands of the job, (WAI 3) Number of current diseases diagnosed by a physician, (WAI 4) Estimated work impairment due to diseases, (WAI 5) Sick leave during the past year (12 months), (WAI 6) Own prognosis of work ability 2 years from now, and (WAI 7) Mental resource [40, 41]. The trial-adapted version consists of 5 items relating to WAI 1, 2, and 4. WAI 4 topic was split into two items that record productivity losses and work quality separately. Items referring to WAI 1 and 4 use a numerical analog scale from 1 to 10; items related to WAI 2 use a 5-point Likert scale with response categories ranging from “poor” to “excellent”. In this trial, the adapted WAI indicators are not evaluated as a score, but as single-item distributions. The described questions on work ability are part of all questionnaires T0 to T3 administered throughout the trial.

*Well-being* is measured via the World Health Organization’s 5-item questionnaire on well-being (WHO-5). The concept of the WHO-5 is based on the Major Depression Inventory, which maps the WHO/ICD-10 criteria for depression. The questionnaire consists of 5 items on general psychological well-being aspects over a 2-week reference period via 6-point Likert scale with response categories ranging from “At no time” to “All of the time”. Between 0 and 5 points are given for each answer. The individual values are added up and multiplied by 4, resulting in a score range of 0–100. Higher values indicate higher well-being. The WHO-5 shows good psychometric properties [42–44]. The WHO-5 also showed a strong negative association with depression symptoms, especially in the range of mild to moderate symptom severity, and is therefore suitable for screening [45]. The WHO-5 is only used in T1 questionnaire.

*Shortness of breath* is measured via 3 specific items using a numerical analog scale from 1 to 10 with pre-COVID-19 and post-COVID-19 values resulting in a total of 6 values. They assess shortness of breath at rest, during light physical activity, and during heavy physical activity. Scores will not be calculated from this. Questions on the shortness of breath are part of the T0, T2, and T3 questionnaires.

*Medication, vaccination status, concentration issues, and COVID-related medical history* are surveyed as single items in order to enable fine-grained subgroup analyses and adjustment for influencing variables. Items on medical history use a numerical analog scale from 1 to 10 with pre-COVID-19 and post-COVID-19 values, if specific symptoms are asked. Scores will not be calculated from this. They are only asked in the T0 baseline questionnaire.

##### Health trackers

Participants from the high-intensity intervention and moderate-intensity intervention groups have health trackers in the form of sliders as a visual analog scale on their overview pages in the LMS. These are labeled as “energy level,” “overall health,” “pain,” “mood,” “amount of enjoyable activities,” and “exercise level” and can be set and saved as often as desired. This offers the possibility of chronological documentation, which allows conclusions to be drawn about the variance of the values referring to the mentioned domains in relation to the individual progress within the therapy program.

##### User statistics

By registering on the LMS, participants agree to their user activity being recorded. The corresponding data is also stored in the participant’s respective data record.

##### Generating data sets

At the end of the trial, all raw values regarding the abovementioned secondary outcome data acquired via the LMS are exported by the platform provider as a data set in .json format (JavaScript Object Notation) and sent to the study management team for evaluation using the end-to-end encrypted virtual data safe. These data sets are pseudonymized without exception. They do not contain any unencrypted personal data.

#### Plans to promote participant retention and complete follow-up {18b}

Participant retention plans are consistent with the strategies to improve adherence to interventions described in {11c}. Full follow-up is usually ensured by adherence to the GP, unless patients generally change their GP. As many of the planned outcomes as possible are collected for all participants, including those who drop out.

#### Data management {19}

The MiLoCoDaS study has a comprehensive data protection conception, including a data protection impact assessment, technical and organizational measures, records of processing activities, and data processing agreements with involved service providers. Data quality assurance has been sufficiently implemented as a requirement of the data protection laws by numerous measures that can be found in the respective documents.

#### Confidentiality {27}

##### General data protection regulations

Throughout the entire project, the data protection regulations according to the GDPR are strictly adhered to and controlled. An accompanying assessment by the responsible department for data protection has been carried out for all relevant aspects. For concrete details on individual measures, please refer to the comprehensive respective documents.

Nevertheless, special reference is made to the constant pseudonymization of patient data at all trial steps.

##### Enrolment and accompanying care

All measures carried out by the GP are subject to medical confidentiality and the data protection standards according to which medical practices are obliged to work. GP keep the pseudonymization key in the patient record, as they are the only party usually holding unencrypted patient data.

##### LMS

Identifying data and health data are stored in different data sets in the LMS database. All health data is stored in pseudonymized form only. Only top-level system administrators of the LMS service provider have direct access to the database. No one else has direct access during or after the study period. However, direct database access during the project period is usually not required. Data exports for analysis exclusively occur in pseudonymized form. The data will be completely anonymized as soon as the purpose and progress of the research permits.

##### Data transfer

Any data transfer between stakeholders that includes the health data of participants will only be conducted in pseudonymized form. When data sets or outcome information is sent, this is done exclusively via end-to-end encrypted password-protected virtual data safe access.

### Plans for collection, laboratory evaluation, and storage of biological specimens for genetic or molecular analysis in this trial/future use {33}

Not applicable, as no biological samples are collected in the MiLoCoDaS study.

### Statistical methods

#### Statistical methods for primary and secondary outcomes {20a}

Generalized linear models will be used to compare the distribution of primary outcomes (sick leave days) in the randomization groups. Link function will be determined based on the observed sample distribution (we expect Poisson or Negative Binomial distribution). Secondary outcomes will be analyzed by Repeated Measurements Analysis of Variance and Mixed Models for Change analysis. Longitudinal response curves will be visualized by graphs showing the marginal estimates in the randomization groups. All statistical analysis will be conducted using the software package STATA (StataCorp. 2019. Stata Statistical Software: Release 16. College Station, TX: StataCorp LLC).

#### Interim analyses {21b}

Interim analyses will only take place in the form of the regular monitoring of participation numbers by the study management team and will not constitute a study termination.

The only defined criterion for study termination is data protection incidents (especially if there is a risk of recurrence or expansion). The final decision on study termination is then made by the primary sponsor.

#### Methods for additional analyses (e.g., subgroup analyses) {20b}

Subgroup analyses will be conducted repeating the efficacy analyses in subgroups of the study population defined by patient-specific variables. Subgroup analyses focus on elucidating observed heterogeneity of effect sizes rather than testing for statistical significance, as the sample size of the study has been defined to achieve sufficient power for the main analysis. Subgroup analyses have only exploratory objectives and nature; therefore, we refrain from adjustment for multiple comparisons. Rather than reporting statistical inference test results, we will report observed effect sizes and related confidence intervals without adjustment for multiple testing.

#### Methods in analysis to handle protocol non-adherence and any statistical methods to handle missing data {20c}

Statistical analysis will be conducted using complete case analysis and as a sensitivity analysis using imputed data based on a multiple imputation algorithm. If the distribution of the observed missing pattern is at random, the core model will be based on the imputed data.

#### Plans to give access to the full protocol, participant-level data, and statistical code {31c}

Unlimited public access is not planned. Following the guidelines of the administering study register, there exists an IPD (Individual Participant Data) Sharing plan for the exchange of participant data. According to its provisions, only aggregated, anonymized data and aggregated questionnaire scale values will be shared. Eligible recipients are researchers who submit a methodologically sound proposal. This applies equally to the dissemination of complete protocols or statistical code. Requests should be directed to the principal investigator via email.

### Oversight and monitoring

#### Composition of the coordinating center and trial steering committee {5d}

The study center is responsible for study coordination. It works closely with the project partners. The responsibilities are distributed as follows:

##### Primary Sponsor (Joachim Fischer)

The Primary Sponsor (PS) is responsible for the coordination and supervision of the team. He also has the final decision on content and conceptual matters.

##### Principal Investigator (Adrian Krotz)

The Principal Investigator (PI) provides oversight for all aspects of the study and the implementation of the intervention.

##### Scrum Master (Stephanie Bechtel)

A Scrum Master is established and responsible for project management. She supports the team to achieve the project’s goals by following the Scrum framework.

##### Conception team (Nadia Sosnowsky-Waschek / Joachim Fischer and coworkers)

Together with the project partners from psychology and physiotherapy, the Primary Sponsor forms the conception team, which is largely responsible for the development of the interventions' content. This team is supported by a group of at least 10 students from these disciplines.

##### Study management team

Principal Investigator, Scrum Master, and other intermittently involved scientific staff form the study management team. The study management team contributes to the development of content in interventions as well as the design and technical implementation of the LMS. It is responsible for the collaboration with healthcare networks and provides ongoing support. In cooperation with the study statistician, it is also responsible for data analysis. The study management team meets on a weekly basis to oversee the study progress and to ensure the integrity of the protocol and conduct of the study.

#### Composition of the data monitoring committee, its role, and reporting structure {21a}

The implementation of a data monitoring committee is not planned for this study. Because of data aggregation, all data will only be obtained in summary form at the end of the study. Only the monitoring of patient allocations is carried out on a regular basis via the LMS study management dashboard.

#### Adverse event reporting and harms {22}

All participants are instructed to report adverse events and harms or side effects at any time to their GP as the first instance. Such events can in addition be reported directly to all other stakeholders in the project. The reporting chain and the cooperation in such cases are defined in the respective contracts.

#### Frequency and plans for auditing trial conduct {23}

There is no audit plan for this study. In particular, since a data analysis only takes place at the end and no adjustments are planned in the meantime, an interim audit is not necessary.

#### Plans for communicating important protocol amendments to relevant parties (e.g., trial participants, ethical committees) {25}

If significant changes to the protocol occur, relevant parties are informed as follows:

*Participants:* Participants receive a system message providing all necessary information via the integrated message function of the LMS. In addition, an email notification is sent about the receipt of the system message.

*Project stakeholders:* If they are affected by the changes, LMS service providers and healthcare networks are notified directly by the study management team via email or phone. GPs receive the information through their affiliated networks.

*Registries, journals, and other external recipients:* The study management team determines the necessary scope of change information at its weekly meeting and initiates appropriate notification.

#### Dissemination plans {31a}

It is planned to publish all trial results as journal articles. Results are additionally to be summarized in brief form on the study center’s website. All participating GPs and healthcare networks will be sent references to results as soon as they are published. For the information of participants, a highly abbreviated layperson’s summary of the results will also be issued to the GPs. Participants can ask their GP about the status of the results at any time.

## Discussion

The primary aim of the present study is to investigate the difference between three modalities of digitally presented 12-module or 12-week psychological intervention with additional medical, physiotherapeutic, and nutritional components for Long COVID patients with respect to possible reduction in sick leave from work during a 180-day follow-up. The intervention addresses patients with mild to moderate severity of Long COVID from primary care practices or occupational health services—patients who occur in large numbers and are currently overwhelming the existing facilities in the health care system. As the three modalities differ regarding costs and scalability, we randomly allocate patients to a fully digital interactive intervention (medium intensity, rapid scalability) or a digital interactive intervention with an additional once-weekly expert webinar. We defined the latter as the intervention of high intensity, at the cost of less rapid scalability due to required expert recruitment and training. The control is augmented information, which mimics material an extensively searching layperson would find on the Internet.

The composition and content design of the individual modules of the program were based primarily on the scientific knowledge available at the start of the study conception in 2022 regarding the manifestation of symptoms in post-COVID patients and available treatment options. At the time of study conception, there was a lack of an evidence-based treatment for post-COVID syndrome—a scarcity that presently continues (autumn 2023). Yet, several guidelines regarding symptomatic treatment of Long COVID have been published. Given the positive experience with digitized interventions in other mental and somatic disorders, we decided to develop an equally symptom-guided and theory-based intervention for Long COVID. Most of the content was primarily derived from methods of Cognitive Behavioral Therapy, supplemented by interventions from physiotherapy, medicine, and nutritional science. To our knowledge, this is the first study of its kind specifically addressing the population of patients suffering from mild to moderate persisting Long COVID symptoms—individuals, who are not fully incapacitated, yet too sick to have fully recovered to resume their original work ability and quality of life.

It should be noted that the MiLoCoDaS study does not aim to provide insights into the causes and pathways of the various functional impairments observed in Long COVID patients. However, the study will provide dense new observational data regarding the courses of certain symptom entities. Further, the recorded meta-data of the LMS platform use and questionnaires will allow exploratory analyses related to patient education in specific coping strategies. This may provide a better insight into the efficacy of psychological support and interventional measures for specific subtypes of the Long COVID condition. At the same time, potential synergistic combinations of measures could be identified. By constantly referring to the potential ability to work and perceived health-related quality of life, the most important socio-economic factors affecting individuals as well as the burden to society are addressed. If shown to be yielding even small effect sizes, due to the high prevalence of patients and the scalability of digital interventions, this program therefore has the potential to relieve the burden on the healthcare system and be cost-effective.

The easy accessibility and scalability of the concept are crucial for the healthcare system and in that respect for society in general. Existing services for the care of Long COVID patients, such as Long COVID outpatient clinics and self-help groups, are usually concentrated in densely populated urban areas, whereas similar services are difficult to implement and rarely found in rural regions. A major challenge is therefore to provide adequate care for affected patients in structurally weak or rural areas. The location-independence of the digital concept is a key advantage in this context. In the future, this could improve the widespread provision of care for people suffering from Long COVID.

One core question is to what extent can psychological online intervention influence the post-viral manifestation of symptoms and help patients regain their ability to work? Considering the WHO’s definition of health as not only the absence of disease or infirmity, but also as a state of complete physical, mental, and social well-being, the biopsychosocial model on which the definition is based is an obvious choice for understanding post-COVID-19 condition as a disorder model. The clinical picture of Long COVID includes a wide range of physical, psychological, and social symptoms and is accompanied by a significant decrease in well-being. In this respect, it can be assumed that the Long COVID symptom bundle undergoes a significant overall improvement of the general state of health through systematic treatment of the main areas of the disorder. Which of the 12 thematically different modules, or which combination of them, can most accurately map a specific mechanism of action is therefore of major interest. On the other hand, it is known from many studies on the treatment of chronic physical illnesses that adjuvant psychological therapy and social support can be highly effective in alleviating somatic complaints. It will be interesting to investigate the effect of the social support provided by the psychological coach and the webinar group in the high-intensity intervention arm compared to the simple availability of the learning content through the LMS.

Of course, it is nevertheless possible that the program is not perceived as individualized or broad enough, despite the wide range of content. This could lead to disappointment, excessive demands, and dropout and negatively impact potential effects.

### Limitations

The number of chosen questionnaires to address aspects of role functioning in daily activities is restricted. We specifically chose the selected constructs to provide a comparison to existing cohorts of healthy adults in the working age from the same population and area. We have previously extensively used the SF-12, which yields subscales on role functioning and impairment. The WHO-5 is an established instrument indicating overall well-being, while the WAI questionnaire addresses aspects of well-being and functioning in the context of work. During the conceptualization, we considered including further specific questionnaires, e.g., on activities of daily life with a focus on self-care or leisure/play in addition. For example, we discussed instruments such as the Barthel scale or the Lawton and Brody IADL assessment. Adequate instruments, however, often require external observation or are not yet validated for use as a self-report. Moreover, because there were clear overlaps with the survey instruments already selected, we aimed to avoid redundancies. Assuming that adherence could be one of the main problems of the study design, being parsimonious in questionnaires was imperative to reduce the risk of additional drop-outs. Therefore, and focusing on the psychosocial approach, we decided against using specific ADL/IADL questionnaires.

The integration of a therapeutic and a preventive approach in the inclusion criteria also involves the risk of varying adherence and dropout. The motivation of already chronically ill patients to make every effort to get well is expected to be much higher than the efforts of acutely ill patients to not necessarily develop Long COVID. Yet, in general, most illnesses such as back pain or depression benefit from early intervention. Moreover, the public perception and also the diagnostic handling of COVID-19 in the healthcare system are currently changing in the expiring pandemic situation. Many cases remain undiagnosed due to the changed guidelines and therefore evade the inclusion criteria in the prevention criterion. In our experience, the overall awareness of the problem is decreasing and similarly the motivation to take preventive measures. The perception of Long COVID as a serious disease varies widely among the population and it is known that only a subset of those infected with SARS-CoV-2 develop a post-COVID-19 condition. Finally, it is generally difficult to show the effectiveness of preventive measures. All this could lead to an underrepresentation of the preventive approach.

A fundamental challenge lies in the expected heterogeneity of the patient sample. Health programs typically benefit from a target group-specific approach. Different levels of education, different experiences with the use of digital media, and their acceptance can influence the effects. Since MiLoCoDaS specifically addresses a cross-section of the population, this perspective could not be taken into account. The intention is to represent conditions as they are found in routine patient care, to lead to the development of a realistic therapy scheme. Therefore, exploratory secondary analyses have been formulated. The bottom line for society is whether such digital intervention and the efforts related to this are cost-effective. Experts in occupational medicine rendered a reduction in sick leave by more than half a week on average as a relevant improvement warranting to include the intervention into the therapeutic armature [46]. We approximated this from the available data as a 4-day reduction at an average duration of 30 days with a standard deviation of 10 days.

Although we specifically designed this study for patients seeking help in primary care practices, we are well aware that occupational therapy and occupational reintegration play a major role in the care of these patients. However, we first wanted to identify the optimum modality of delivery and obtain insight on the utility of the individual modules and their specific content. Taking the occupational aspect to a greater extent into account would have resulted in a possible lack of focus in the research question. It therefore seemed more appropriate to examine the general efficacy of the different program modalities in this step, in order to then revise and tailor this digital intervention more to the specific needs of occupational reintegration, embracing concepts of occupational therapy in a possible further step.

Another possible issue in the program design is that several psychological contents are introduced relatively extensively. This was required by the study design, which only provided for a conversation with a psychologically trained coach for one group. All content, including the basics of coping with worries, fears, pain, and depressive moods, which can be assigned to psychotherapy, is otherwise worked out by the participants themselves. Overall, this aspect can also reduce the acceptance of the coaching.

### Possible conceptual risks

A major risk might be unblinding. Through interaction between participants from the same population area or targeted enquiries at their GP, participant could get an impression of specificities of the different study arms and thus bypass blinding. Unblinding might lead to disappointment in patients in the control arm, which in turn might exaggerate possible treatment effects. However, due to the wide catchment area for this study, the participation of numerous different GPs and the purely digital application, we consider this risk to be reduced, but it cannot be ruled out. Further, the exclusion criteria do not cover every imaginable special situation that could jeopardize blinding. For example, a couple who both suffer from Long COVID and have the same GP could participate in the trial in accordance with the rules and be assigned to different groups. However, adding less likely special cases to the already extensive inclusion and exclusion criteria would further complicate the process and thereby possibly discourage more physicians from enrolling patients. Finally, we cannot rule out non-specific effects of a group-webinar session that are content independent. This could be addressed in a future trial comparing unstructured webinar group session to the content of MiLoCoDaS.

The fact that the study design provides for enrollment via GPs has advantages and disadvantages. On the one hand, the inclusion and exclusion criteria are automatically checked by qualified specialists. In addition, the participants can use familiar structures and have a known contact person for questions. Furthermore, it is the only way to realistically test the setting of using a digital-only intervention like the MiLoCoDaS LMS integrated into the GP’s treatment routine, which strengthens the reliability of derived statements on feasibility.

On the other hand, the enrollment must be integrated into the routine processes of the GP practices. Although the organization is facilitated by the top-down structure via the primary care networks, the final implementation of the enrollment measures depends on the time capacities of the practice. It also requires initial onboarding of the respective practices for process orientation. Many practices and GPs may shy away from the effort of this procedure and therefore refrain from arranging enrollment appointments. This could make it more difficult to reach sufficient participant numbers.

At the same time, there is a risk of bias, if only particularly motivated GPs who are aware of the problem enroll patients. Patients of GPs, who do not accept Long COVID as an independent clinical condition or who do not consider it to be of significance, will not be given access.

There are numerous dropout risks for successfully enrolled patients. Due to the digital-only form of the program, which offers advantages for accessibility and scalability, there is the disadvantage of a lack of human support and contact. This applies in particular to the control and the moderate intensity intervention group, which have no regular human support, meaning that an impending drop-out cannot be recognized or counteracted.

It is also possible for participants to drop out silently by simply not logging in anymore. Although the LMS sends automatic reminder emails, these also lack the option of responding individually to the respective reasons for leaving.

For the high-intensity intervention group, there is a specific dropout risk due to the initial waiting period. After enrolment, registration in the LMS, and randomization, participants in this group must first select a webinar group. Once this has been done, the first webinar and therefore access to the platform content only starts when the required group size has been reached. If only a few participants are enrolled or randomly assigned to the high-intensity intervention group in a given period, these waiting times can quickly become quite long. This could lead to a large number of dropouts. It may therefore be necessary to flexibly adjust the minimum group size to the current enrollment rate in order to counteract mass dropout.

Overall, the structure of the trial may be very sensitive to possible deviations from the planned sample size and predicted adherence rates.

## Conclusion

Although the study design is facing some limitations and challenging issues and not all patients will probably benefit to the same extent also due to the broad variety of symptoms they suffer from, the educational digital intervention offers an opportunity to sufficiently augment physicians’ arsenal in caring for this group of patients.

### Trial status

The recruitment of participants started at the end of March 2023. The recruitment should be completed by the end of October 2023. The current protocol version is 2.3 of 2023-03-01.

### Supplementary information

**Additional file 1.** SPIRIT checklist

## Data Availability

Access to the final data set will be given to the study team of the Medical Faculty Mannheim, Heidelberg University, Center for Preventive Medicine and Digital Health (CPD), Division of General Medicine, and the study team of the project partner SRH University of Applied Sciences Heidelberg. There is no restriction for investigators.

## Abbreviations

ADL: Activities of daily living
AMBIGOAL-ANCOR: *AMBulante Integrierte Gesundheitszentren zur Optimierung der Ärztlichen Versorgung und Pflege im Ländlichen Raum - ANwendungserweiterungen COvid-19 Restitutionsphasen*
BEM: German structured employment reintegration program for employees with long-term diseases (original: *Betriebliches Eingliederungsmanagement*)
CBI: Copenhagen Burnout Inventory
CMDQ: Cornell Musculoskeletal Discomfort Questionnaire
COVID-19: Coronavirus disease 2019
DMHI: Digital mental health intervention
DiGA: Digital health applications (German: *Digitale Gesundheitsanwendung*)
ENRICHD: Enhancing Recovery in Coronary Heart Disease study
ESSI: Social-Support subscale according to the ENRICHD-study
FFB-MOT: Questionnaire for the assessment of physical function status
GAD-7: Generalized Anxiety Disorder questionnaire
GP: General practitioner
IADL: Instrumental activities of daily living
IPD: Individual Participant Data
json: JavaScript Object Notation
LMS: Learning Management System
ME/CFS: Myalgic encephalomyelitis/chronic fatigue syndrome
MiLoCoDaS: Mild to moderate Long COVID digital intervention study
OI: Orthostatic intolerance
p.a.: Per annum (per year)
PEM: Post-exertional malaise (PEM)
PHQ-9/PHQ-15: Patient Health Questionnaire, 9 or 15 item Subscales
PI: Principal Investigator
PROMs: Patient-reported outcome measures
PS: Primary Sponsor
SARS-CoV-2: Severe acute respiratory syndrome coronavirus type 2
SD: Standard deviation
SF-12: Short-form 12
SMD: Standardized mean difference
WAI: Work Ability Index
WHO-5: WHO Well-being questionnaire.
